# Knockdown of Amyloid Precursor Protein in Zebrafish Causes Defects in Motor Axon Outgrowth

**DOI:** 10.1371/journal.pone.0034209

**Published:** 2012-04-24

**Authors:** Ping Song, Sanjay W. Pimplikar

**Affiliations:** Department of Neurosciences, Lerner Research Institute, Cleveland Clinic, Cleveland, Ohio, United States of America; University of North Dakota, United States of America

## Abstract

Amyloid precursor protein (APP) plays a pivotal role in Alzheimer’s disease (AD) pathogenesis, but its normal physiological functions are less clear. Combined deletion of the APP and APP-like protein 2 (APLP2) genes in mice results in post-natal lethality, suggesting that APP performs an essential, if redundant, function during embryogenesis. We previously showed that injection of antisense morpholino to reduce APP levels in zebrafish embryos caused convergent-extension defects. Here we report that a reduction in APP levels causes defective axonal outgrowth of facial branchiomotor and spinal motor neurons, which involves disorganized axonal cytoskeletal elements. The defective outgrowth is caused in a cell-autonomous manner and both extracellular and intracellular domains of human APP are required to rescue the defective phenotype. Interestingly, wild-type human APP rescues the defective phenotype but APPswe mutation, which causes familial AD, does not. Our results show that the zebrafish model provides a powerful system to delineate APP functions *in vivo* and to study the biological effects of APP mutations.

## Introduction

Amyloid precursor protein (APP) has been a focus of intense investigation because of its central role in Alzheimer’s disease (AD) pathogenesis [1]–[3]. It is a ubiquitously expressed, single-pass transmembrane protein that is constitutively processed into multiple smaller fragments [4]. Of these, the large ectoplasmic soluble APPα fragment (sAPPα; generated by α-secretase) exerts neurotrophic effects, the smaller amyloid-β (Aβ) peptides (products of β- and γ-secretases) have been shown to play a pivotal role in AD pathogenesis, while APP Intracellular domain (AICD), a product of γ-secretase activity, regulates intracellular signaling and likely also contributes to AD pathogenesis [5], [6].

Less is known about the normal physiological functions of APP compared to its pathological role in AD [7], [8]. Previous *in vitro* studies suggested that APP plays a role in cell migration and neuronal extension [9]. At this time, the functions of APP are not completely understood and studies have produced conflicting results [8], [10]–[12]. We previously showed that knockdown of APP in zebrafish results in convergent-extension defects [13]. Most of the *in vivo* functional analyses of APP have been performed in transgenic mice that lack APP or the combination of APP and APP-like protein 2 (APLP2). Deletion of APP alone does not cause lethality, but the animals show a series of more subtle phenotypic defects such as reduced grip strength, stunted growth, decreased brain weight deficits, and impaired LTP [14]–[16] that can be rescued by sAPPα [17]. Consistent with theses results, the extracellular domain of APL-1, an APP homologue in *Caenorhabditis elegans*, rescues the defects in *apl-1* mutants [10]. However, combined deletion of APP and APLP2 results in postnatal lethality and a recent study showed that sAPPβ (soluble ectoplasmic peptide produced by BACE cleavage) failed to rescue the lethality and neuromuscular synapse defects in double-KO mice [11].

The observation that APP/APLP2 double-KO mice display postnatal lethality indicates that APP performs an important but redundant function during embryogenesis. Here, we used a zebrafish (*Danio rerio*) model system to dissect the functional role of APP. Zebrafish embryos develop *ex vivo,* enabling direct observation of the developing embryo in real time. In addition, zebrafish offer a number of advantages over mouse models, including the large number of eggs produced and the ability to modulate protein expression by antisense morpholino (MO) [18]–[20]. In this study we report that knockdown of zebrafish APPb caused defects in embryonic development and inhibition of axonal outgrowth of the facial branchiomotor neurons Vp and VII and spinal motor neurons in a cell-autonomous manner. Interestingly, we found that only full-length APP (human APP_695_ or zebrafish APPb) but not truncated forms, rescued the defective phenotypes, indicating that both extracellular and intracellular domains of APP are required for its normal functions.

**Figure 1:**
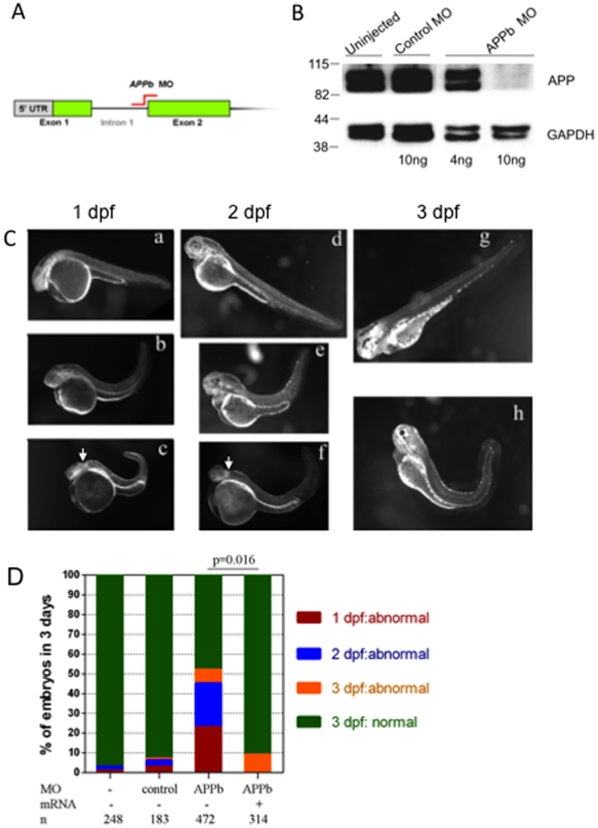
APPb is required for normal zebrafish embryonic development. (A) Schematic representation of APPb-MO blocking the mRNA splicing site between intron 1 and exon 2 (indicted in red) as used in this study. (B) Western blot analysis of APPb protein in zebrafish embryos at 2 dpf. The lower panel was probed with anti-GAPDH antibody. At 2 dpf, APPb protein migrated as a doublet at 98 KDa with stronger expressions in the un-injected group and control MO group. With the injection of 10 ng of APPb MO per embryo, APPb protein levels were not detected at 2 dpf. With the injection of 4 ng of APPb MO per embryo, weak expression of APPb protein migrating at 98 KDa was observed. (C) Morphological features of control embryos (a, d, g) and APPb morphant embryos (b, c, e, f, h). Lateral views (anterior to the left and dorsal at the top) of zebrafish embryos. The gross anatomical phenotype included a deformed body and a shortened and curved tail. In addition, defects in midbrain patterning were observed (arrows). 1 dpf: a, b, c; 2 dpf: d, e, f; 3 dpf: g, h. (D) APPb mRNA rescues the defective phenotype. There is little effect on normal embryonic development caused by the injection of control morpholino (APPb mis-match MO). Zebrafish embryos injected with 10 ng of APPb-MO expressed abnormal phenotypes at the 1 dpf (red), 2 dpf (blue) and 3 dpf (yellow) developmental stages. Embryos co-injected with 10 ng of APPb-MO and 350 pg APPb mRNA expressed normal phenotypes during embryogenesis. Statistical significance was measured comparing APPb-MO embryos and the co-injected embryos (p = 0.016, p<0.05, in 2-tailed paired *t* tests).

## Results

Zebrafish express two gene products, termed APPa and APPb, which are homologues of the human APP gene [21]. APPb, a 694-residue protein, more closely resembles the human APP_695_ isoform, which is selectively expressed in the brain. Previously we showed that knockdown of APPb by antisense MO resulted in impaired embryonic development, resulting in shortened body axis, deformed tail and small eyes [13], which resembled a defective convergence-extension phenotype. In the present investigation, we studied the effects of APPb knockdown at the cellular level and examined motor neuron axonal outgrowth.

**Figure 2:**
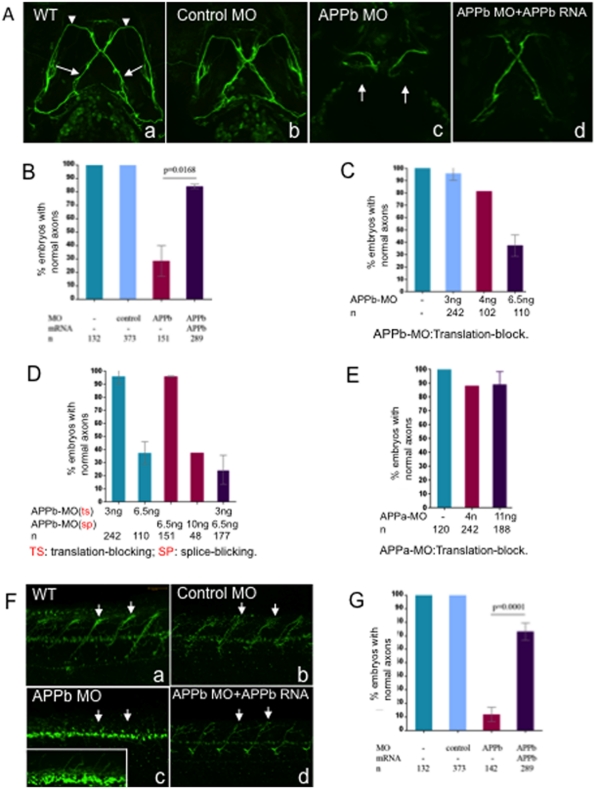
Embryos co-injected with APPb-MO and APPb mRNA rescued defective phenotypes of the Vp and VII and spinal cord nerve projections observed in APPb morphants. (A) Knockdown of APPb disrupts the projections of axons Vp and VII (3 dpf) when injected with APPb MO. Uninjected embryos and embryos injected with control MO did not show altered axonal outgrowth of neurons Vp and VII. Embryos injected with APPb-MO expressed axonal inhibition of axons Vp and VII (arrows). The defective phenotype was rescued by co-injection of APPb-MO and APPb mRNA. Ventral view; anterior, top. (B) Quantification of embryos expressing normal axonal outgrowth of neurons Vp and VII rescued through co-injection of APPb-MO (splice-block) and APPb mRNA. Uninjected embryos and embryos injected with control MO showed normal axonal growth of Vp and VII neurons. Embryos co-injected with 10 ng of APPb-MO and 350 pg APPb mRNA rescued the defected phenotype observed in the APPb morphant group. Statistical significance was established between APPb-MO embryos and co-injected embryos (p = 0.0168, p<0.05, in 2-tailed paired *t* test). (C) The translation-block MO of the APPb caused the identical defected phenotype on the axonal outgrowth of the Vp & VII neurons as the splice-block MO of the APPb; both were dose-dependent. (D) The morphants showed an identical defected phenotype on axonal outgrowth of Vp & VII neurons when co-injected with the translation-block MO (3 ng per embryo) of the APPb and the splice-block MO (6.5 ng per embryo) of the APPb at lower doses that did not produce a defective phenotype individually. (E) There was no defective phenotype of axonal outgrowth of Vp & VII neurons in the APPa morphants when injected with the translation-block MO against APPa. (F) APPb function is required for normal nerve outgrowth of the spinal cord. Lateral views of 3 dpf embryos (anterior is to the left, dorsal is at the top). Uninjected embryos and control embryos expressed normal motor nerve projections from the spinal cord to the myotomes (5–10 somites). Embryos injected with 10 ng of APPb-MO (splice-block) expressed severe motor neuron axon defects, including aberrant projections and decreased branching (arrows pointing at axon). Embryos co-injected with APPb-MO and APPb mRNA rescued the severe phenotype observed in the morphant group. The white rectangle in (c) is an amplification of the spinal cord neurons, which shows branching defects of the neurites in the APPb morphants. (G) Downregulation of APPb affects normal projection of motor nerves in the spinal cord. Compared with the control MO and uninjected groups, embryos injected with 10 ng APPb-MO (splice-block) showed significant axonal inhibition. Embryos co-injected with 10 ng of APPb-MO and 350 pg of APPb mRNA rescued the APPb morphant phenotype. Statistical significance was observed between morphant embryos and co-injected embryos (p = 0.0001, p<0.05 in 2-tailed paired *t* test).

### Knockdown of APPb Results in Abnormal Zebrafish Development

We injected zebrafish embryos at the one cell stage with APPb morpholino (APPb-MO) directed at the mRNA splicing site between intron 1 and exon 2 (Figure 1A, Figure S1) or directed at the translation initiation site [13] in order to reduce APP levels throughout the entire embryo. To ensure the effectiveness of APP knockdown, total protein extracts from embryos at 2 days post-fertilization (2 dpf) were separated by SDS-PAGE and probed with an antibody raised to the C-terminal 15 residues of human APP (the last 15 residues are identical in both species). There was a dose-dependent decrease in APP protein levels in APPb-MO-injected embryos compared to uninjected or control-MO-injected embryos (Figure 1B). By contrast there were no significant changes in levels of GAPDH, which was used as a loading control. These results indicate that APPb-MO inhibited the expression of APPb for at least 48 hours post-injection.

We examined control and treated embryos morphologically every 24 hours for up to 3 dpf (Figure 1C). Injection of 10 ng APPb-MO caused defective development, resulting in embryos with deformed body shapes, shortened and curved tails and atrophied midbrains compared to uninjected or control-MO-injected embryos. The morphant phenotype was apparent in some APPb-MO-injected embryos as early as 1 dpf (compare panels b and c vs. a, Figure 1C). Approximately 25% of APPb-MO-injected embryos, which appeared normal morphologically at 1 dpf, displayed the defective phenotype at the 2 dpf or even at the 3 dpf stage, whereas less than 5% of control-MO-injected or uninjected embryos showed aberrant development at these stages (panels d-h). Some of the APPb morphants that shown normal in morphology at the 1 dpf displayed abnormal morphology at the 2 dpf or 3 dpf. The precise reason why some embryos showed delayed morphant phenotype is not completely clear. However, it is possible that small variations in the amount of injected morpholino may account for the appearance of phenotype at later days. Quantification from multiple separate experiments showed that injections of 10 ng APPb-MO caused approximately 50% of embryos to show defective development by 3 dpf (Figure 1D), whereas less than 10% of the control-MO injected embryos showed a defective phenotype at this stage. At 1 dpf about 25% of APPb-MO-injected and less than 5% of control-MO-injected embryos were defective (red columns in Figure 1D). At 2 dpf, an additional 20% of APPb-MO-injected embryos had developed the abnormal phenotype (blue columns; see also arrows in panels c and f of Figure 1C) and another 5% of embryos displayed abnormal phenotypes at 3 dpf (orange columns). To study the specificity of this effect, we co-injected mRNA encoding zebrafish APPb with APPb-MO and observed that the defective phenotype was almost completely prevented by co-injecting 350 pg of APPb mRNA (last column in Figure 1D). Injection of the same amount of mRNA alone did not affect morphological development (Table S1) or axonal growth (Table S2), suggesting that there was no toxicity associated with injecting this level of APPb mRNA. These data indicate that the developmental abnormalities were caused specifically by reduced APPb levels. Together, these observations suggest that APPb function is required for normal embryonic development in zebrafish.

**Figure 3:**
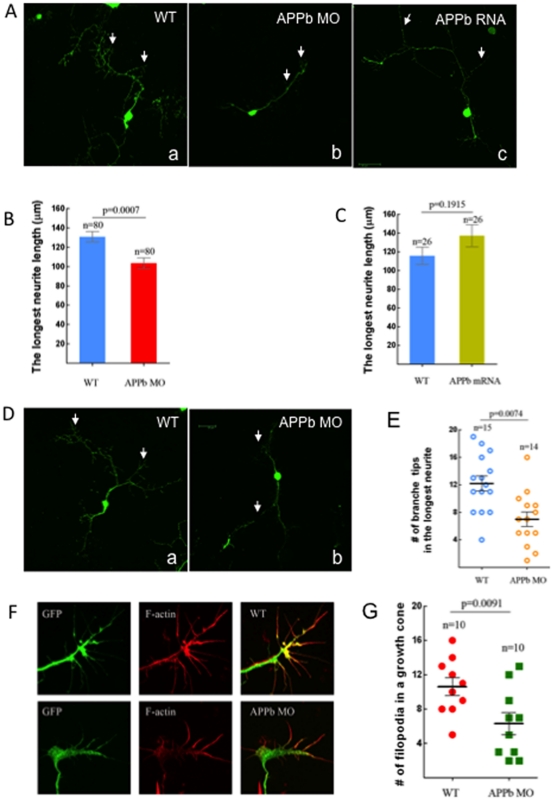
Neuronal cell cultures derived from embryos injected with APPb-MO had decreased neurite length. (A) Neuronal culture from control embryos and APPb mRNA (350 pg) over-expression embryos exhibited on average 3 primary branches with several secondary branches. Neuronal cultures from embryos injected with 8 ng of APPb-MO exhibited on average 1 primary branch with many secondary branches. 8 ng of APPb-MO is sufficient to inhibit neruite growth, so embryos were injected with 8 ng of the APPb MO instead of the 10 ng to limit the potential for toxicity. The neurons were cultured for 2 days before fixing. (B) Down-regulation of APPb in cultured neurons affected normal neurite growth. The neurons from the embryos injected with 8 ng of APPb-MO showed a decrease in neurite length compared to neurons from the control embryos (un-injected). The average neurite length of a control neuron was about 130 µm, compared to only about 100 µm in the morphant embryos. Statistical significance was observed between control neurons and APPb knockdown neurons (8 ng APPb MO) (p = 0.0007, p<0.05 in two-tailed paired *t-test*). (C) Quantifying the effects of APPb mRNA in cultured neurons. No significant difference was observed in neurite length between control and APPb mRNA over-expression cultured neurons. Control cultured neurons expressed a shorter neurite length compared to APPb mRNA over-expression neurons. No statistical significance was observed (p = 0.1915, p>0.05 in two tailed paired *t-test*). (D) There were fewer branches of neurites in the APPb knockdown neurons (8 ng of APPb MO) than in control neurons (WT); only branches with more than 5 µm were counted. The neurons were cultured for 2 days. (E) Down-regulation of APPb in cultured neurons affected the number of branch tips. Compared to control neurons, APPb-MO cultured neurons showed a decreased number of branch tips of the longest neurite. Statistical significance was observed between control and APPb-MO cultured neurons (p = 0.0074, p<0.05 in two tailed paired *t-test*). (F) Down-regulation of APPb in cultured neurons affected the morphology and projection of growth cones and filopodia. APPb knockdown neurons expressed abnormal growth cone morphology and a decreased number of filopodia (20 hour neuron cultures). (G) Reduced number of filopodia in APPb knockdown cultured neurons. Compared to control cultured neurons, APPb-MO (8 ng per embryo) cultured neurons expressed a reduced number of filopodia. Statistical significance was observed between control and APPb cultured neurons (p = 0.0091, p<0.05 in two tailed paired *t-test*).

### APPb is Required for Normal Motor Axon Outgrowth During Zebrafish Development

To study the effects of APPb knockdown at the cellular level, we examined the axonal outgrowth of motor neurons in *Tg(isl1:gfp)* fish, which express GFP in facial branchiomotor and spinal motor neurons [22], [23] (kindly provided by Dr. Anand Chandrashekhar). To minimize the potential for confounded results from severe developmental defects (the APPb morphants that shown severe defects in body morphology had defective growth of the axons), we analyzed only those APPb morphant embryos that showed normal morphology or only mild morphological abnormality. APPb-MO- or control-MO-injected embryos were allowed to develop for 3 days and the midbrain region was examined from the ventral side. Uninjected or control-MO-injected embryos showed the characteristic cross-wire pattern of nV and nVII (arrowheads and arrows respectively; Figure 2A, panel a). However, injection of APPb-MO resulted in almost complete inhibition of nVII axonal elongation and significant inhibition of nV elongation (arrows in Figure 2A, panel c). Quantification across multiple experiments showed (Figure 2B) that injection of APPb-MO caused a morphant phenotype in approximately 70% of embryos (n = 151; Figure 2B). Uninjected (n = 132) embryos and embryos injected with control MO (n = 373) showed normal axonal elongation (Figure 2A, panels a, b). We further corroborated the specificity of the morphant phenotype by using translation-block MO [13], which also produced a similar phenotype in a dose-dependent manner (Figure 2C). The specificity of the MO effect is further confirmed by the demonstration that sub-threshold doses of both MOs, neither of which produced a morphant phenotype when injected alone, caused defective axonal elongation when simultaneously administered (Figure 2D). Interestingly, APPa MO [13] had no effect on axonal elongation (Figure 2E). However, APPa MO affected migration of the VII neurons (data not shown) indicating that APPa MO were effective in our assay system but did not affect axonal elongation. The results above show that APPb function is specifically required for proper elongation of faciobrachial motoneurons.

**Figure 4:**
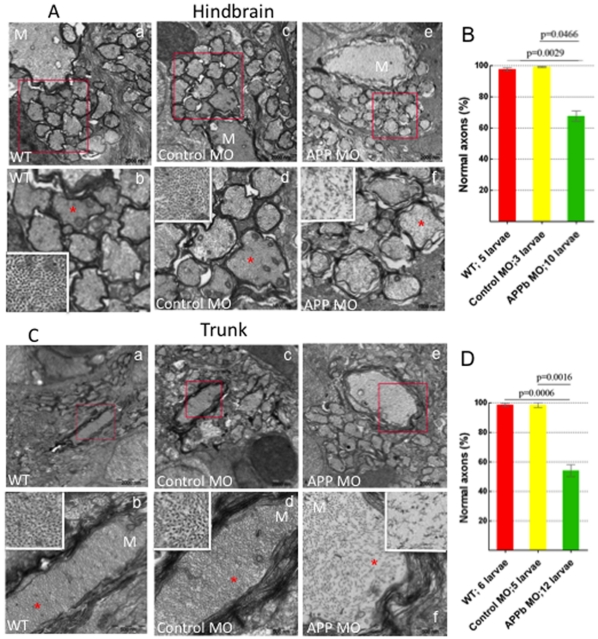
Analysis of Transmission Electron Microscopy images of axons in zebrafish hindbrain and trunk regions (5 dpf). (A) Transmission Electron Microscopy (TEM) images of axons in zebrafish hindbrain. Compared to uninjected and control MO-injected embryos, zebrafish embryos injected with APPb-MO (8 ng) expressed a decreased density and disorganization of the cytoskeleton in both the Mauthner (M) axons and the axons around the M axon. Panels b, d, and f are amplifications of the boxed areas in panels a, c and e, respectively. The white rectangles in panels b, d, and f are amplifications of the areas marked with red 5-point stars. (B) Quantifying the defects of axonal cytoskeletal morphology in the hindbrain of APPb morphant embryos. At 5 dpf, zebrafish embryos injected with 8 ng of APPb-MO showed a disruption in axon cytoskeletal dynamics in the hindbrain region. For the TEM experiment, the embryos were injected with 8 ng of the APPb MO instead of 10 ng. The embryos that were injected with 8 ng of the APPb MO experienced disorganization of the axonal cytoskeleton; the cytoskeleton of the embryos that were injected with 10 ng of APPb MO experienced severely defects in or loss of axons. Statistical significance was observed comparing uninjected (263 axons) and morphant (662 axons) larvae (p = 0.0029, p<0,05 in two-tailed paired *t-test*). Statistical significance was also observed between control MO (217 axons) and morphant larvae (p = 0.0466, p<0.05 in two tailed paired *t-test*). (C) TEM images of axons at in the zebrafish trunk section. Compared to uninjected and control MO-injected larvae, zebrafish larvae injected with 8 ng of APPb-MO expressed a decrease in axonal density and had defects in cytoskeletal organization of axons, including the M axon. Panels b, d, and f are amplifications of the boxed areas in panels a, c and e, respectively. The white rectangles in panels b, d and f are amplifications of the areas marked with red 5-point stars. (D) Quantifying defects of axonal cytoskeletal morphology in the trunk region of APPb morphant embryos. At 5 dpf, zebrafish embryos injected with 8 ng of APPb MO expressed an abnormal phenotype in axonal cytoskeletal organization in the trunk region. Statistical significance was observed comparing uninjected (217 axons) and morphant (562 axons) larvae (p = 0.0006, p<0.05 in two tailed paired *t-test*). Statistical significance was also observed between control MO (212 axons) and morphant larvae (p = 0.0016, p<0.05 in two tailed paired *t-test*).

To further establish that the observed abnormal phenotypes were caused specifically by the knockdown of APPb, we co-injected APPb-MO with mRNA encoding APPb in embryos at the one cell stage and observed that in a vast majority of embryos, co-injection with APPb mRNA rescued the abnormal phenotype (Figure 2A, panel d; Figure 2B, n = 289). In addition, human APP695 was also able to rescue the morphant phenotype (see the result).

We also examined the axonal outgrowth of spinal cord motor neurons located in the trunk region in the middle of each spinal cord hemi segment [24]. The uninjected embryos (n = 132) and those injected with control-MO (n = 373) showed normal development, with motor neuron axons projecting from the spinal cord to the myotomes (Figure 2F, panels a, b; Figure 2G). However, greater than 90% of embryos injected with APPb-MO exhibited axonal projections that were shorter and less branched (n = 142; arrows in panel c in Figure 2C; Figure 2G). The severity of the abnormal phenotype was dose-dependent (data not shown). When co-injected with APPb mRNA, less than 25% of morphant embryos showed spinal cord motor axon inhibition (panel d in Figure 2F; Figure 2G), indicating that the phenotype was due to specific knockdown of APPb.

**Figure 5:**
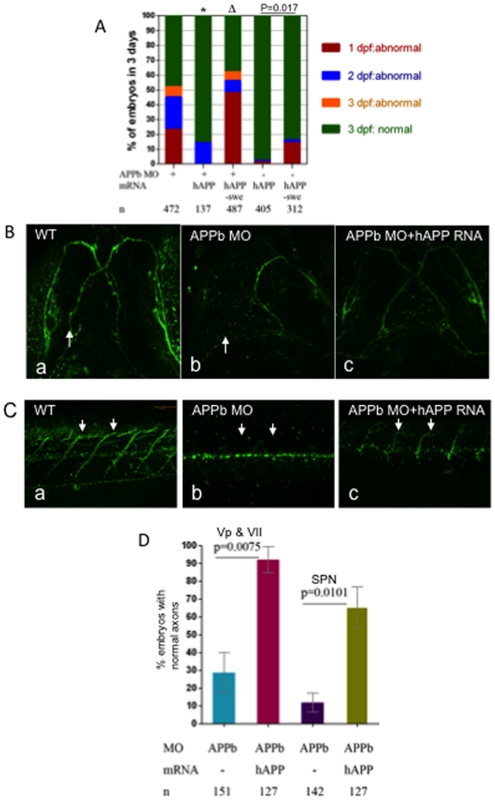
hAPP_695_ (hAPP), but not hAPP-swe, effectively rescued defects in axonal outgrowth in APPb-MO morphants. (A) Co-injection of hAPP_695_ mRNA rescued APPb morphant embryonic morphology. As depicted, embryos were inspected over a period of 3 days. Co-injection of full length human APP_695_ mRNA rescued the defective phenotype observed in the APPb morphant embryos (*, p = 0.026, p<0.05 in two tailed paired *t-test*). In contrast, human APP-Swedish mutation (Δ, p = 0.5241, p>0.05) and AICD mRNA failed to rescue the APPb morphant phenotype. Combined injections (10 ng of APPb-MO and 350 pg of AICD mRNA) caused more severe deficits compared to injecting APPb-MO alone. (B) Co-injection of hAPP_695_ mRNA rescued axonal outgrowth of motor neurons Vp and VII of the APPb morphant. Zebrafish embryos co-injected with full length human APP_695_ rescued the APPb morphant axonal outgrowth phenotype of motor neurons Vp and VII (arrow). (C) Embryos co-injected with hAPP_695_ rescued the defective phenotype of motor axons in the spinal cord in APPb morphant embryos. Uninjected embryos expressed normal motor neuron projections from the spinal cord to the myotomes (5–10 somites). Embryos injected with (10 ng) APPb-MO expressed severe motor neuron axonal defects including aberrant projections and decreased branching. Embryos co-injected with APPb-MO and hAPP_695_ mRNA had a rescued phenotype compared to the morphant group. Lateral views of 3 dpf embryos (anterior is to the left, dorsal is at the top). (D) Quantification of embryos expressing normal axonal outgrowth of neurons Vp and VII and spinal cord rescued through co-injection of hAPP695 mRNA. Injection of 10 ng of APPb-MO caused abnormal axonal outgrowth of motor neurons Vp and VII in 75% of embryos. Co-injection with mRNA encoding full-length human APP695 overwhelmingly rescued the APPb morphant phenotype (p = 0.0075, p<0.05 in two tailed paired *t*-test) (3 dpf). Embryos injected with 10 ng APPb-MO showed significant inhibition of spinal cord axon outgrowth. Embryos co-injected with 10 ng of APPb-MO and 350 pg of hAPP695 mRNA rescued the APPb morphant phenotype (p = 0.0101, p<0.05 in two tailed paired *t*-test).

Together, these data provide compelling evidence that APPb is required for normal axonal outgrowth of facial branchiomotor neurons Vp and VII as well as spinal cord motor neurons, and a reduction in APP levels caused defective axonal outgrowth, which was rescued by co-injection with APPb mRNA.

### Downregulation of APPb in Cultured Neurons Affects Normal Neurite Growth

APP is a transmembrane protein and undergoes constitutive processing to release cleaved products in extracellular and intracellular compartments. Thus, APPb can act in cell-autonomous or non-autonomous fashions. To examine whether APPb functions in a cell-autonomous or non-autonomous fashion, we prepared neuronal cultures from control or APPb-MO-injected Isl1-GFP embryos. Isl1 promoter is expressed in motoneurons and sensory neurons [22], [23]. Thus, the GFP positive cells in culture (Figure 3) represent both motoneurons and sensory neurons. Neuronal cultures prepared from control embryos (un-injected) exhibited long axons and multiple primary branches (Figure 3A, panel a) with several secondary branches (arrows). In contrast, the neuronal cell cultures derived from embryos injected with APPb-MO exhibited decreased neurite growth (Figure 3A, panel b vs. a) and significantly reduced branching. These neurons had on average one primary branch with many secondary branches (Figure 3A, panel b) and the severity of neurite growth inhibition increased with increasing dosage of APPb-MO (data not shown). The longest neurite length measured in control neurons was 130 µm (n = 80; Figure 3B) whereas the longest in the APPb-MO-injected group was approximately 100 µm (n = 80; Figure 3B). In general the neurites from the APPb-depleted neurons (n = 14) showed fewer branches than the control neurons (n = 15; Figure 3D, E). Since reduced APPb levels caused a decrease in neuritic length, we asked whether increasing APPb levels would have the opposite effect. We injected embryos with mRNA encoding APPb and prepared the neuronal cultures. We found a trend toward neurite length (n = 26) prepared form APPb over-expressing embryos being longer than that in control neurons, but this difference did not reach statistical significance (panel c in Figure 3A; Figure 3C).

**Figure 6:**
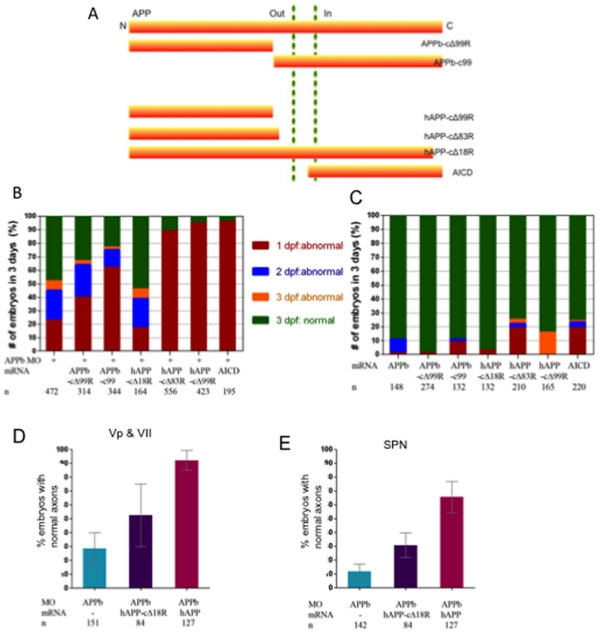
Truncated APP fragments cannot rescue the APPb morphant phenotype. (A) Schematic representation of the full length Amyloid Precursor Protein (APP) and the truncated mutations used in this study. (B) Full length APP is required for normal embryonic development. As depicted by the graph, embryos co-injected with APPb-MO and APPb-cΔ99R or APPb-c99 or hAPP-cΔ18R or hAPP-cΔ83R or hAPP-cΔ99R or AICD mRNA did not rescue the APPb morphant embryos. By contrast, excluding hAPP-cΔ18R, these truncated APP fragments increased the number of abnormal embryos in the APPb morphants. (C) The morphological effects on embryonic development of over-expression of the truncated mutants of APP mRNA (350 pg). The APPb-c99, hAPP-cΔ83R, hAPP-cΔ99R and AICD have limited domain negative effects on embryo development compared with full length APPb and hAPP. (D) Human APP-cΔ18R partially rescued the defective phenotypes of axons Vp and VII observed in APPb morphants compared to full length hAPP. (E) Human APP-cΔ18R partially rescued the defective phenotypes of spinal cord axons observed in APPb morphants compared to full length hAPP.

We also examined the morphology of growth cones and noticed that the growth cones of APPb-depleted neurons were smaller and had a significantly reduced number of filopodia (Figure 3F, G) compared to control neurons. Together theses results suggest that knockdown of APP causes a significant reduction in neurite length and growth cone complexity, and that this effect is most likely cell-autonomous.

### Downregulation of APPb Induces Cytoskeletal Disorganization

Cytoskeletal elements play a crucial role in axonal outgrowth as well as in growth cone formation. The observations that knockdown of APPb resulted in both reduced axonal outgrowth as well as aberrant growth cone morphology prompted us to examine whether these phenotypes were associated with disorganization of the axonal cytoskeleton in APPb morphant embryos. Uninjected, control-MO- or APPb-MO-injected embryos were allowed to grow for up to 5 dpf (Figure S2) and were processed for transmission electron microscopy (EM). We studied the axonal cytoskeleton in the hindbrain (the region of facial branchiomotor neurons; Figure 4A) and the trunk region (to observe spinal cord motor neurons, Figure 4C). To minimize variability in sample preparation for EM, we examined the hindbrain regions from 5 uninjected embryos, 3 control-MO-injected embryos and 10 APPb-MO-injected embryos. Similarly, the trunk region was examined in 6 uninjected embryos, 5 embryos injected with control-MO and 12 embryos injected with APPb-MO. Representative images are shown in Figures 4A and 4C and quantification of the number of morphologically normal neurons is shown in Figures 4B and 4D, respectively. We observed significant differences in the Mauthner (M) axon and the axons centered around the Mauthner axon between the APPb morphants and uninjected or control-MO embryos in both the hindbrain and trunk regions. Whereas the axons from control embryos were tightly packed with microtubules (see insets in panels b and d of Figures 4A, 4C), those from APPb-MO-injected embryos showed a reduced number of microtubules, which were also loosely packed (inset in panel f of Figures 4A, 4C). Thus, the cytoskeletal density in the APPb-MO axons was significantly reduced compared to controls. It should be noted that sensory axons cannot be distinguished from other neuronal axons in EM. We cannot rule out the possibility that other major axonal tracts also show developmental defects upon APPb-MO injections in EM images. Nonetheless, these data show that reduced APPb levels during development resulted in a dis-organized axonal cytoskeleton, which may be responsible for the impaired axonal outgrowth and aberrant growth cones in APPb morphant larvae.

### Human APP_695,_ but not APPswe, Rescued Phenotypic Defects in APPb Morphant Embryos

Since the defective phenotype caused by APPb knockdown could be rescued by expression of APPb (Figure 1), we next asked whether expression of human APP (APP_695_) could also rescue the morphant phenotype. We co-injected APPb-MO embryos with 5′ capped APP_695_ mRNA at the one cell stage and inspected the embryos every 24 hours for 3 days for morphological abnormalities (Figure 5A) and axonal outgrowth of neurons Vp & VII (Figure 5B) as well as spinal cord motor neurons (Figure 5C). Like APPb mRNA, embryos co-injected with APP_695_ mRNA had a rescued morphant phenotype, as over 85% of embryos were found to be normal at the 3 dpf stage when co-injected with APP_695_ mRNA compared to less than 50% when injected with APPb-MO alone (n = 137, Figure 5A). Interestingly, mRNA encoding APPswe mutant protein was unable to rescue the phenotype, as approximately 60% of embryos were found to be abnormal when co-injected with APPswe mRNA compared to approximately 53% embryos injected with APPb-MO alone (Figure 5A). To ensure that the mRNA alone did not cause any significant toxicity, we injected the same amount of mRNA without APPb-MO and observed that APP_695_ mRNA caused no abnormality and that APPswe mRNA injection alone caused approximately 15% of embryos to develop abnormally (last 2 columns in Figure 5A; Table S3, S4, S5).

We extended these observations to nV and nVII outgrowth (Figures 5B, 5D) and spinal motor neurons (Figures 5C, 5D). We observed that injection of APPb-MO alone caused a morphant phenotype, but co-injection with APP_695_ mRNA significantly rescued the morphant phenotype for both sets of motor neurons. APPswe mRNA was much less effective in reversing the defective phenotype (data not shown). Quantification of these data indicated that while only 30% of embryos injected with APPb-MO alone showed normal outgrowth of nV and nVII neurons, co-injection with hAPP_695_ mRNA overwhelmingly rescued the phenotype, with over 90% of embryos developing normally (Figure 5D). Thus, wild-type APP_695_ mRNA was equally effective in rescuing the APPb morphant phenotype in all 3 of our experimental measures: morphological abnormality (as observed by small body, deformed tail etc.), outgrowth of facial branchiomotor neurons, and outgrowth of spinal motor neurons. However, rescue by APPswe was less efficient. Thus, these results collectively indicate that human APP_695_, which shares 69% homology with zebrafish APPb, can functionally replace the latter during embryonic development. Moreover, these results suggest that a lack of APP function can result in multiple phenotypic defects, with certain phenotypes being more sensitive to this change.

### Full-length APP, but not Truncated Constructs, Rescues APPb Morphants

Since full-length zebrafish APPb and human APP695 were able to rescue the developmental defects caused by reduced APPb levels, we next used this system to map the functional domain in APP that was necessary for this rescue. Embryos were co-injected with the indicated truncated APP mRNA constructs (Figure 6A) and were examined every 24 ng hours over 3 days of development. We observed that the extracellular domain of APPb (APPb-cΔ99), which is equivalent to human sAPPβ, was not able to rescue the defective phenotype. Similarly, APPb-C99 peptide was also unable to rescue the defect, indicating that both extracellular and intracellular domains of APPb are necessary for proper APP functioning. Likewise, both human sAPPα and sAPPβ (hAPP-cD83, and hAPP-cD99, respectively in Figures 6A, 6B) were non-functional in this assay. Importantly, deletion of the last 18 residues of human APP was sufficient to render APP non-functional. Finally, free cytosolic AICD, which contains the last 18 residues of APP, was also unable to rescue the morphant phenotype, showing that both extracellular and intracellular domains of APP are required for its full functional activity. Injections of mRNA alone did not produce any significant deleterious effects (Figure 6C). Only, sAPPα and AICD encoding mRNA when injected alone showed moderate toxicity (about 20% abnormal embryos, Figure 6C; see also Figure S3; Table S6, S7).

To further verify these results, we examined the potential of hAPP-cD18R to rescue the outgrowth defects of motor neurons. Again, we noticed that whereas full-length hAPP_695_ was able to rescue up to 90% of embryos injected with APPb-MO (Figure 6D), this number was significantly reduced in the case of hAPP-cD18R. A similar result was observed in spinal motor neuron outgrowth (Figure 6E). Together, our results provide compelling evidence that C-terminal 18 residues are necessary but not sufficient for proper APP functions during embryonic development in zebrafish.

## Discussion

In this study we injected zebrafish embryos with antisense MO to reduce APPb levels throughout the entire embryo and examined live embryos to study embryonic development, axonal outgrowth of facial branchiomotor neurons and outgrowth of spinal motor neurons at different stages of development. Our data show that a reduction in APPb levels produces dramatic developmental defects and results in deformed (morphant) embryos. More importantly, our studies show that APPb function is required for proper axonal outgrowth of motor neurons in zebrafish. To our knowledge, this is a first investigation of the effects of APP knockdown on axonal outgrowth in a live vertebrate embryo.

These studies revealed two important aspects of the biological functions of APP. First, we provide direct evidence that reducing APPb levels results in impaired axonal outgrowth of motor neurons in live embryos. APP belongs to a family of proteins and studies on single APP knockout mice revealed only subtle phenotypes, very likely due to the presence of functionally redundant members of the APP gene family [25]. By contrast APP and APLP2 compound knockout mice show lethality in the early postnatal period [26]. Although the reasons for postnatal lethality are unclear, the findings from mouse models indicate that APP performs an essential function during embryogenesis. However, mouse embryos develop *in utero* and therefore do not provide an optimal model to directly observe developing embryos. In contrast developing zebrafish embryos are readily accessible *ex vivo* for direct visualization. We believe that our findings demonstrate the potential of the zebrafish model to examine APP function during embryogenesis.

The second important conclusion of the present investigation is that both extracellular and intracellular domains of APP are necessary for proper APP function and that the APP mutation (APPswe) that causes familial AD is not able to compensate for the loss of wild-type APP in the zebrafish model. The APP family of proteins is highly evolutionarily conserved and has highly conserved domains in both the extracellular and intracellular regions of APP. Full-length zebrafish APPb and human APP695 were both able to rescue the APPb knockdown morphant phenotype, but none of the truncated mutant proteins were able to do so effectively. We cannot completely rule out the possibility that the truncated APP fragments did not fold correctly or were rapidly degraded and therefore could not rescue the defective phenotype. However, studies in mouse models have also shown that APP fragments are unable perform the functions of full-length APP [11], [27]. Thus, we believe that our data further confirm that both extracellular and intracellular domains of APP are essential for APP function. These observations are consistent with the fact that both domains of APP contain highly conserved motifs. The finding that APPswe was unable to rescue axonal outgrowth defects in motor neurons or to rescue defective development could be of relevance to AD pathology. APPswe undergoes increased β-secretase processing and generates more Aβ peptides [28] and the currently favored amyloid hypothesis suggests that increased Aβ production plays a crucial role in causing AD. Our findings reveal an additional pathological consequence of Swedish mutation, namely the inability of the mutant protein to support normal functioning of APP during embryogenesis.

We provide clear evidence that reducing APPb levels causes impaired axonal outgrowth *in vivo* and future studies are required to understand the underlying mechanism. Our EM data show gross disorganization of cytoskeletal elements in axons of APPb-depleted embryos. The connections between APPb knockdown and cytoskeletal disorganization are unclear but it is likely that APP intracellular domain plays an important role in this phenomenon. AICD is known to exhibit multiple biological effects including the regulation of gene expression involved in cytoskeletal organization [29], [30]. AICD contains the conserved ‘EYNPTY’ motif, which acts as a docking site for multiple cytosolic adapter proteins including Fe65, JIP1, x-11a and others [31], [32]. The finding that the hAPP-cD18R mutant, which lacks only the last 18 residues (including the ‘EYNPTY’ motif), is unable to rescue the APPb morphant phenotype confirms the importance of AICD in APP function. Indeed, a recent study in transgenic mice arrived at similar conclusion [33]. It is important to note that APPb-c99, which does contain the EYNPTY motif but lacks most of the ectoplasmic domain (including both E1 and E2 conserved motifs), is also non-functional in the zebrafish model we describe here. Thus, APP intracellular domain is necessary but not sufficient for the proper functioning of APP.

Finally, we believe that the zebrafish model system used here will be crucial for understanding APP function in vertebrates. C. *elegans* and *drosophila* animal models have generated important insights into APP function, but these invertebrate models have also generated information which is inconsistent with that observed in mouse models [34]. On the other hand, although the information generated from studying knockout mouse models is more relevant to humans (as well as to AD pathogenesis), embryonic development in mice is not amenable to direct examination of live embryos. Moreover, different mouse models have generated inconsistent or contradictory information, perhaps due to genetic complexity [3]. Thus, the zebrafish model occupies a unique niche, as it is relevant to humans as a vertebrate animal and yet it also provides an unmatched *in vivo* system to directly observe events during embryogenesis. Our findings that this model can be successfully used to distinguish the biological activity of APPswe mutant protein from that of wild-type APP provides a powerful demonstration of the potential of the zebrafish model to study familial AD mutations in APP.

## Materials and Methods

### Zebrafish

The Tg (isl 1: GFP) line of zebrafish (*Danio rerio*) [23] was maintained at 28°C under a controlled photoperiod (14 hour light: 10 hour dark cycle). Embryos were collected from natural spawning of adult fish and raised in an incubator at 28.5°C. Embryos and larvae were fixed in 4% phosphate-buffered paraformaldehyde (PFA) (Sigma) at 4°C and stored at 4°C until use for further analysis. Pigmentation of embryos and larvae was inhibited by adding 0.003% PTU (Sigma) to water. All experimental procedures and fish protocols in this study were approved by the Cleveland Clinic Institutional Animal Care and Use Committee.

### mRNA/Morpholino Microinjection

Full-length APPb cDNA (Clone ID 7086881, Open Biosystem) and truncated APPb-c99, APPb-cΔ99 were subcloned into T3TS vector and linearized with BamH I; full-length human APP_695_ (hAPP), hAPPswe (carrying NL mutation), hAPP-CΔ18R, hAPP-CΔ83R, hAPP-CΔ99R (lacking C-terminal 18, 83 or 99 residues, respectively), AICD (containing C-terminal 59 residues) were subcloned into the pCS2+ vector and linearized by Not1 digestion [16]. The mRNA for each gene was transcribed using an mMessage mMachine Kit (Ambion). 350 pg of mRNA was injected at the one-cell stage.

Antisense morpholino oligonucleotides (MOs) (Gene Tools) were designed to target the APPb intron1-exon2 boundary (Figure 1A), 5′ GCACCTGCCAACAGCACAACCGGCA 3′ (APPb MO); a 5 base mismatch morpholino as the control morpholino: 5′ GCAGCTGCGAACACCACAAGCGCCA 3′ (misAPPb MO) (Figure S1); we also used a APPb MO (translation-block) [13] and a APPa MO (translation-block) [13] in this study. Fish embryos at the one-cell stage were injected with the indicated amount of APPb MO or misAPPb MO. For the rescue experiments, the mixture of the APPb MO (10 ng) and mRNA (lacking the morpholino target sequence; 350 pg) was injected at the one-cell stage of each embryo.

### Western Blotting

Proteins were isolated by homogenizing 2 dpf embryos in RIPA buffer (Millipore) containing protease inhibitor cocktail (Sigma). SDS-PAGE and western blotting were performed as described previously [16]. The antibodies used were anti-APP C-terminal antibody 0443 (Calbiochem, 1∶2000) and GAPDH (Chemicon, 1∶4000). Band density was normalized to GAPDH loading controls.

### Live Larvae Confocal Images

Live larvae were anesthetized in a 0.10% tricaine (Sigma) solution in fish water and then mounted in a 1% low-melt agarose 0.10% tricaine solution. Larvae were placed on glass slides under coverslips and were imaged using a Leica-SP5 Confocal Microscope.

### Whole-mount Immunofluorescence

Larvae were euthanized in 0.10% tricaine anesthetic, fixed in 4% PFA at 4°C overnight at the designated dpf, treated with collagenase (1 mg/mL in water) at 25°C for 30 min and bleached in 3% hydrogen peroxide and 1% KOH for 30 minutes. After washing in PBS (pH 7.4) with 0.01% Triton-X100 (PBT), larvae were incubated in blocking buffer (5% BSA in PBS, with 0.01% Triton-X100) with 1∶500 diluted primary mouse anti-GFP antibody (N86/38, 75–132; UC Davis/NIH Neuromab Facility Antibodies Incorporated) overnight at 4°C. After washing with 0.01% Triton-X100 in PBS, this was followed by 1∶500 diluted Alexa-Fluor-488 goat anti-mouse conjugated secondary antibody (Invitrogen, A-11029). The larvae were mounted with a 1% low-melt agarose and imaged with a confocal microscope with excitation lasers at 488 nm (Fluorescein).

### Neuron Cultures and Immunostaining

GFP-marked neurons were obtained from the Tg (isl 1: GFP) line of zebrafish embryos. Embryos developed at 24–26°C and were collected after 24 hours. Following washing with sterile water, the embryos were treated with 70% ethanol for 3 seconds and then washed 3 times with Neurobasal medium (Gibco, 21103, no l-Glutamine) containing 100 u/ml penicillin, 100 µg/ml streptomycin, 2 µm/ml B-27 supplement (GIBCO), and 28 mM L-Glutamine. Approximately 50 larvae were deyolked by puncturing the yolk sac with a 100 µl pipette tip and then washed by suspending them in medium 3 times. The washed larvae were dissociated by passing the 10 µl tip 100 times and then sat for 5 minutes before collecting the supernatant. The same procedure was repeated 3 times. The cells (neurons) in the combined supernatant were filtered with a 40 µm Nylon filter (BD Falcon, 352340) and then transferred into a COSTAR 24 well cell culture cluster (3524) that was pre-coated with poly-D-lysine. The cultures were plated at a cell density of 30–50 embryos per well. The cultures were maintained at 25°C for 2 or 3 days. Following the removal of the medium, neurons were fixed in 4% PFA at 4°C overnight. The cultured neurons were washed with PBS with 0.01% Triton-X100 3 times and then were incubated with blocking buffer (5% BSA in PBS with 0.01% Triton-X100) for 2 hours at 25°C. The mouse anti-GFP antibody (1∶500 dilution) was used as primary antibody incubated overnight at 4°C. Thereafter, the cultured neurons were washed 3 times with 0.01% Triton-X100 and then incubated with Alexa-Fluor-488-conjugated secondary antibody (1∶500 diluted) at 4°C for overnight. The stained neurons were imaged by fluorescence microscopy (Leica DM5500B) or confocal microscopy.

### Axonal Length Quantification

The stained cultured neurons were visualized under a Leica fluorescent microscope with attached mercury light bulb source and filter cubes. GFP was visualized in the green channel. Neurons were randomly selected and images were taken via the Leica Microsystems_AP software program in the green channel. Axonal length was measured by tracing the longest axon of each imaged neuron. Measurements were properly recorded and documented.

### Transmission Electron Microscopy (TEM) Analysis

Larvae were collected at 5 dpf (days post fertilization), anesthetized with tricaine (Sigma), and then fixed with fresh 4% formaldehyde in 1% glutaraldehyde in PBS at 4°C overnight. The larvae were transferred to 1% osmium tetroxide and dehydrated using a graded ethanol series followed by treatment with propylene oxide and embedded in Epon-812 resin. Ultra-thin sections (50–60 nm) were mounted on grids and post-stained with 3% uranyl acetate in 50% ethanol and 1% lead citrate in 0.1 M sodium hydroxide and imaged under a Philips Tecnai G2 20 S-TWIN microscope.

### Statistical Analysis

Statistical analysis (paired *t* tests) was performed using the software package GraphPad Prism Software. Differences were considered to be significant with a *p*-value <0.05.

## Supporting Information

Figure S1 **The information about the design of the APPb morpholino blocking the mRNA splicing site between intron 1 (indicted in black) and exon 2 (indicted in red).**
(TIF)

Figure S2 **Embryos injected with APPb-MO (splice-block) still expressed motor neuron axon defects at 5 dpf.**
(TIF)

Figure S3 **Morphological features of normal (a, c, e) and abnormal (b, d, f) embryos injected with APP mRNA and its truncated mRNA.**
(TIF)

Table S1 **The embryos injected with the 350** **pg of the zebrafish APPb mRNA expressed the normal morphology.**
(TIF)

Table S2 **The embryos injected with the 350** **pg of the APPb mRNA were normal on axon growth.**
(TIF)

Table S3 **About 95% of the embryos injected with 220** **pg–350** **pg of the hAPP695 mRNA were normal.**
(TIF)

Table S4 **There was no toxicity on the embryo development when injecting 350** **pg of the hAPP695 mRNA per embryo.**
(TIF)

Table S5 **The embryos injected with the 350** **pg of the hAPP695 mRNA expressed the normal axon growth.**
(TIF)

Table S6 **The embryos injected with 350** **pg of the hAPP-**Δ**18R mRNA were normal on morphology.**
(TIF)

Table S7 **The embryos injected with 350** **pg of the hAPP-**Δ**18R mRNA expressed the normal axon growth.**
(TIF)
