# Supplementary figures and images for: Knockdown of Amyloid Precursor Protein in Zebrafish Causes Defects in Motor Axon Outgrowth
Source: PLoS One. 2012 Apr 24;7(4):e34209. doi: 10.1371/journal.pone.0034209 (PMC3335837; doi:10.1371/journal.pone.0034209)

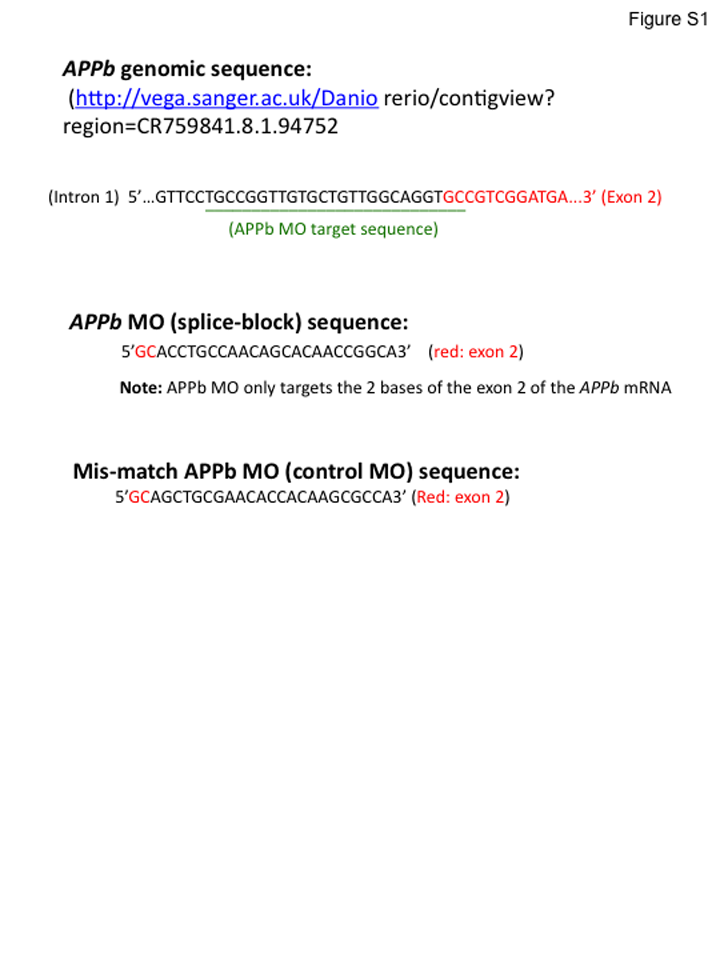

Supplement: Figure S1 — The information about the design of the APPb morpholino blocking the mRNA splicing site between intron 1 (indicted in black) and exon 2 (indicted in red). (TIF) [file pone.0034209.s001.tif]

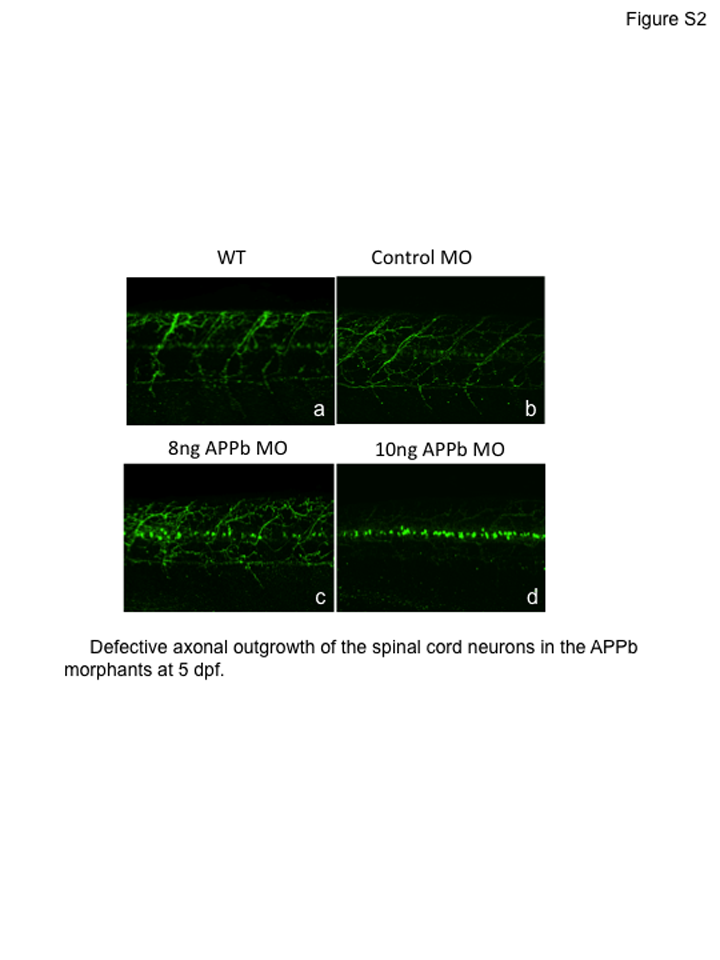

Supplement: Figure S2 — Embryos injected with APPb-MO (splice-block) still expressed motor neuron axon defects at 5 dpf. (TIF) [file pone.0034209.s002.tif]

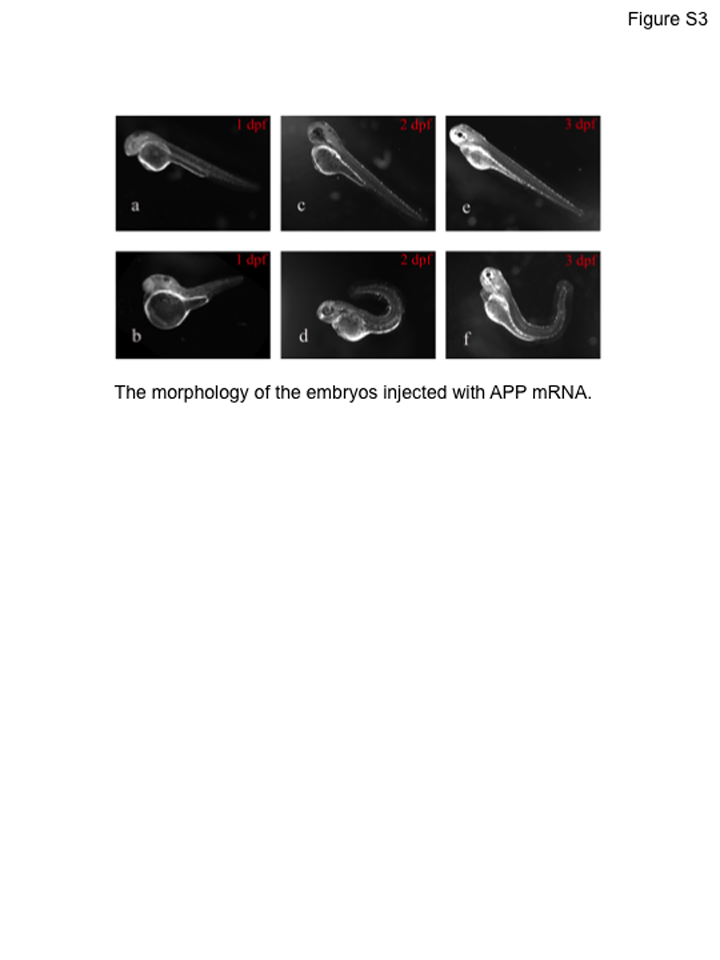

Supplement: Figure S3 — Morphological features of normal (a, c, e) and abnormal (b, d, f) embryos injected with APP mRNA and its truncated mRNA. (TIF) [file pone.0034209.s003.tif]

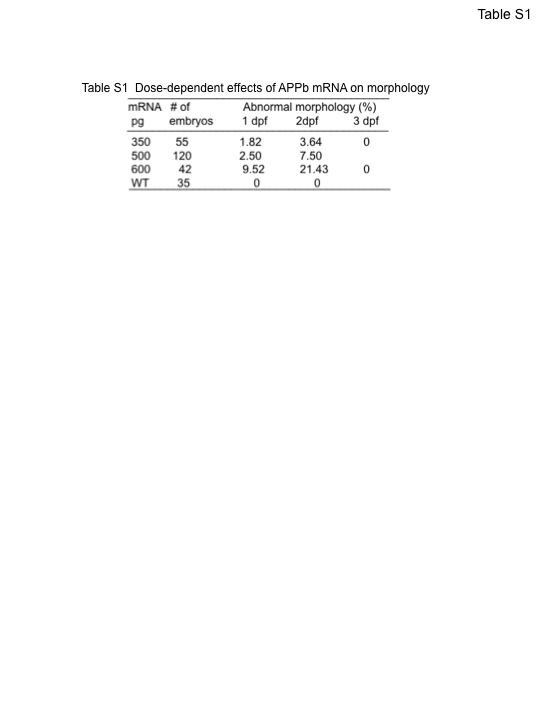

Supplement: Table S1 — The embryos injected with the 350 pg of the zebrafish APPb mRNA expressed the normal morphology. (TIF) [file pone.0034209.s004.tif]

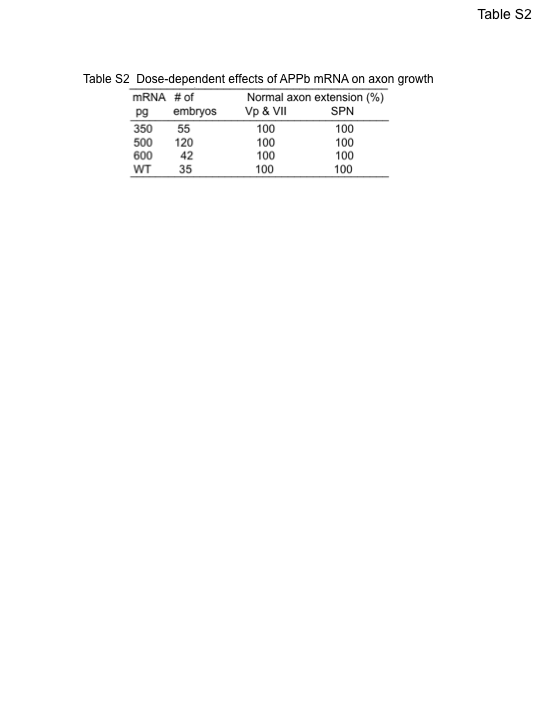

Supplement: Table S2 — The embryos injected with the 350 pg of the APPb mRNA were normal on axon growth. (TIF) [file pone.0034209.s005.tif]

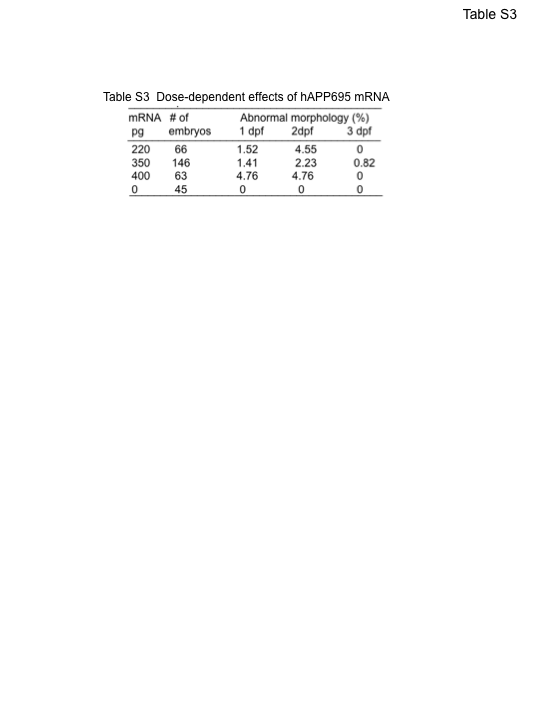

Supplement: Table S3 — About 95% of the embryos injected with 220 pg–350 pg of the hAPP695 mRNA were normal. (TIF) [file pone.0034209.s006.tif]

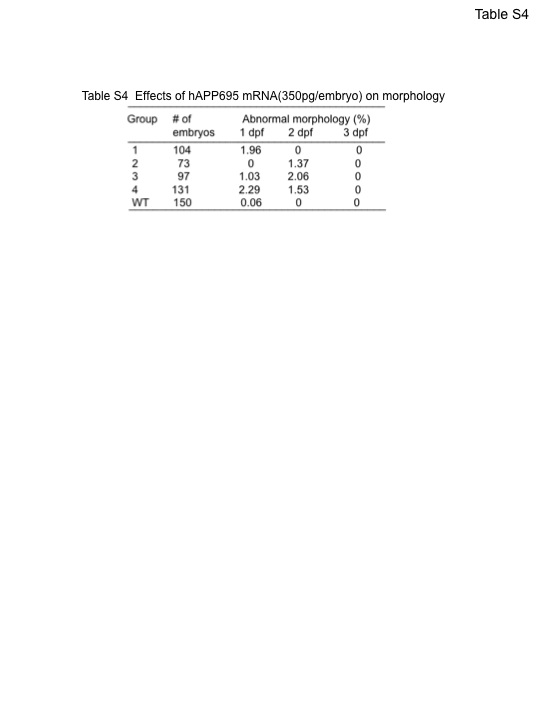

Supplement: Table S4 — There was no toxicity on the embryo development when injecting 350 pg of the hAPP695 mRNA per embryo. (TIF) [file pone.0034209.s007.tif]

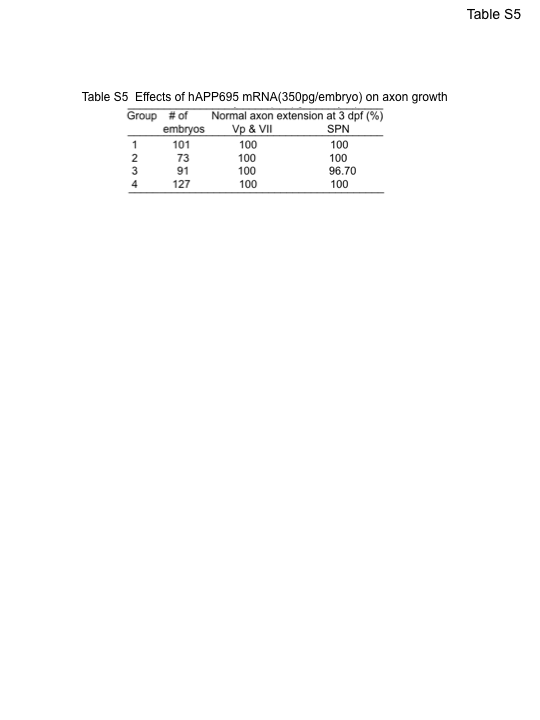

Supplement: Table S5 — The embryos injected with the 350 pg of the hAPP695 mRNA expressed the normal axon growth. (TIF) [file pone.0034209.s008.tif]

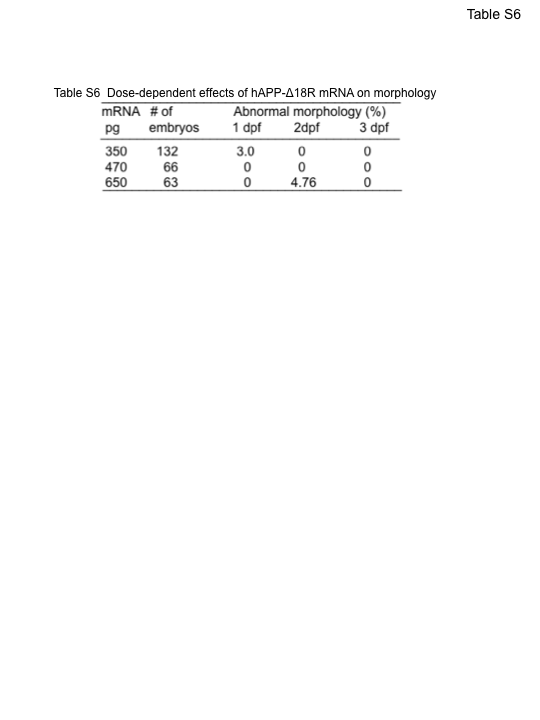

Supplement: Table S6 — The embryos injected with 350 pg of the hAPP-Δ18R mRNA were normal on morphology. (TIF) [file pone.0034209.s009.tif]

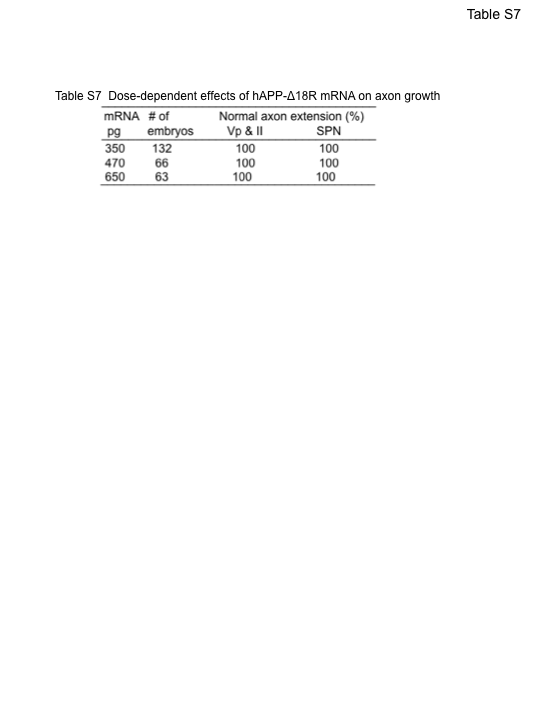

Supplement: Table S7 — The embryos injected with 350 pg of the hAPP-Δ18R mRNA expressed the normal axon growth. (TIF) [file pone.0034209.s010.tif]
